# Draft Genome Sequences of Three Lactic Acid Bacterial Strains Investigated for B Vitamin Biosynthesis

**DOI:** 10.1128/mra.00144-23

**Published:** 2023-05-30

**Authors:** Lu Gao, Gyu-Sung Cho, Erik Brinks, Rohtraud Pichner, Charles M. A. P. Franz

**Affiliations:** a Department of Nutritional, Food, and Consumer Sciences, University of Applied Sciences Fulda, Fulda, Germany; b Department of Microbiology and Biotechnology, Max Rubner Institute, Federal Research Institute of Nutrition and Food, Kiel, Germany; University of Rochester School of Medicine and Dentistry

## Abstract

The draft genome sequences of three lactic acid bacteria, namely, Limosilactobacillus reuteri 92071, Lactiplantibacillus plantarum 92117-i3, and Limosilactobacillus fermentum 92072, and the presence of genes involved in the biosynthesis of B vitamins were determined. Limosilactobacillus reuteri 92071 showed complete gene clusters for vitamin B_12_ biosynthesis, with a GC content of 38.52 mol%.

## ANNOUNCEMENT

Vegan nutrition and vegetarianism have become popular due to rising consumer interest in healthier and more sustainable lifestyles, but they carry the risk of B vitamin (particularly vitamin B_12_) deficiency (1). Biofortification of vitamin B_12_ through microbial fermentation is a potential tool to improve vitamin deficiency in elderly individuals and vegans (2). Several strains of lactic acid bacteria (LAB) have been found to be cobalamin producers and are receiving much attention as potential starter cultures (3); however, levels of vitamin B_12_ production by LAB species in such foods are often not sufficient to meet the required daily intake. Moreover, no significant evidence that these bacteria are able to produce the active vitamin B_12_ form was found (4). Therefore, we conducted whole-genome sequencing of different LAB species from our culture collection, which had been originally isolated from fermented food using de Man, Rogosa, and Sharpe (MRS) agar, to investigate their cobalamin synthesis-related genes, as well as relevant genes for the synthesis of other B vitamins.

Three strains were cultivated in MRS broth (VWR, Darmstadt, Germany) at 37°C for 24 h and presumptively identified by 16S rRNA gene sequencing. The total genomic DNA was extracted using the peqGOLD bacterial DNA isolation kit (PeqLab, Erlangen, Germany) according to the manufacturer’s instructions. The TruSeq Nano DNA library preparation kit was used to prepare the genomic DNA library. The MiSeq reagent kit was used for 2 × 250-bp paired-end sequencing with the MiSeq platform, according to the manufacturer’s instructions (Illumina, Munich, Germany). The raw sequencing data were trimmed using Trimmomatic v. 0.32 (parameters: Phred33, sliding window:4:15, leading:3, minlen:45) (5) and *de novo* assembled using SPAdes v. 3.13.2 (parameters: –careful, –k 21,33,55,77) (6). All contig sequences that were shorter than 500 bp or contaminated with PhiX sequence were removed using the BBDuk pipeline with default parameters (DOE Joint Genome Institute).

Orthologous average nucleotide identity (ANI) and *in silico* DNA-DNA hybridization (DDH) values were calculated with the most closely related type strains (Table 1) using the Orthologous ANI Tool (OAT) v. 0.93.1 and the Genome-to-Genome Distance Calculator (GGDC) server from DSMZ with formula 2.

**TABLE 1:** *De novo* assembly of three LAB strains and draft genome features, including genes for cobalamin, folate, and riboflavin biosynthesis

Parameter	Finding for^*a*^:		
	Lactiplantibacillus plantarum 92117-i3	Limosilactobacillus reuteri 92071	Limosilactobacillus fermentum 92072
No. of contigs	34	148	149
No. of paired sequence reads	621,803	428,913	593,872
*N*_50_ (bp)	242,008	32,962	24,975
GC content (mol%)	44.62	38.52	52.19
Total length (bp)	3,180,242	2,201,984	1,806,298
Genome coverage (×)	48	46	78
No. of coding sequences	2,956	2,244	1,812
No. of tRNAs	53	62	52
No. of rRNAs	5	5	4
No. of pseudogenes	35	48	104
ANI (%)^*b*^	99.13 (Lactiplantibacillus plantarum)	98.47 (Limosilactobacillus reuteri)	97.33 (Limosilactobacillus fermentum)
DDH (formula 2) (%)^*b*^	93.3 (Lactiplantibacillus plantarum)	88.2 (Limosilactobacillus reuteri)	78.0 (Limosilactobacillus fermentum)
Cobalamin biosynthesis gene(s)			
*cob* genes	*cobB*, *cobQ*	*cobB*, *cobC*, *cobD_1*, *cobD_2*, *cobQ_1*, *cobQ_2*, *cobS*, *cobT*, *cobU*	*cobB*, *cobQ*
*cbi* gene(s)	*cbiO*	*cbiA*, *cbiC*, *cbiD*, *cbiE*, *cbiF*, *cbiG*, *cbiH*, *cbiJ*, *cbiK*, *cbiL*, *cbiM*, *cbiN*, *cbiO*, *cbiQ*, *cbiT*	ND^*c*^
*hem* genes	*hemA*, *hemH*	*hemA_1*, *hemA_2*, *hemB*, *hemC*, *hemL*, *hemH*	ND
Folate biosynthesis genes	*folA*, *folB*, *folC*, *folD*, *folE*, *folK*, *folP*, *folT_1*, *folT_2*	*folB*, *folD*, *folE*, *folP*, *folT*	*folB*, *folC*, *folD*, *folE*, *folP*
Riboflavin biosynthesis genes	*ribBA*, *ribD*, *ribE*, *ribF_1*, *ribF_2*, *ribH*	*ribB*, *ribF*	*ribB*, *ribF*
GenBank accession no.	JAQOWJ010000000	JAQOWD010000000	JAQOWE000000000
BioSample accession no.	SAMN32886983	SAMN32886977	SAMN32886978

a All annotation information was derived from the BV-BRC only for general function prediction.

b Species identification with respect to the most closely related type strain (indicated in parentheses). Species-level cutoff values were 96% for ANI and 70% for DDH, according to previous studies (7, 8).

c ND, not detected.

The *N*_50_ values were between 24,975 and 242,008 bp, and the genome coverage ranged from 46- to 78-fold (Table 1). All contigs were annotated using the Bacterial and Viral Bioinformatic Resource Center (BV-BRC) v.3.28.9 tools, prokka (v. 1.12) and the NCBI Prokayotic Genome Annotation Pipeline (PGAP) v. 6.4 with default parameters. The three draft genome sizes ranged from 1.80 Mbp to 3.18 Mbp, with GC contents between 38.52 and 52.19 mol% (Table 1).

All three strains show a potential for B vitamin biosynthesis, particularly Limosilactobacillus reuteri 92071, which showed the presence of complete gene clusters for vitamin B_12_ biosynthesis. Lactiplantibacillus plantarum 92117-i3 and Limosilactobacillus fermentum 92072 possessed only partial genes involved in cobalamin biosynthesis (Table 1). These strains could be potential starter culture candidates for production of vitamin B_2_ and vitamin B_9_, since they possess the core subset of the known genes for biosynthesis of these vitamins.

### Data availability.

The draft genome sequences and the raw read data were deposited in DDB/ENA/GenBank under the accession numbers listed in Table 1.
